# Survival Benefit of Adjuvant Radiotherapy After Surgery in Patients With T1‐2N1M0 Hypopharyngeal Squamous Cell Carcinoma: A Dual‐Cohort Analysis of SEER and Institutional Data

**DOI:** 10.1002/cam4.71555

**Published:** 2026-01-30

**Authors:** Zhangwei Hu, Renqiang Ma, Yihui Wen, Jie Deng, Wei Sun, Lin Chen, Siyu Chen, Weiping Wen, Wenbin Lei

**Affiliations:** ^1^ Department of Otolaryngology, the First Affiliated Hospital Sun Yat‐Sen University Guangzhou Guangdong People's Republic of China; ^2^ Otorhinolaryngology Institute Sun Yat‐Sen University Guangzhou Guangdong People's Republic of China; ^3^ Department of Otolaryngology, the Sixth Affiliated Hospital of Sun Yat‐Sen University Guangzhou China

**Keywords:** hypopharyngeal squamous cell carcinoma, overall survival, postoperative radiotherapy, SEER, systemic therapy

## Abstract

**Background:**

The optimal therapeutic strategy for patients with T2‐3N0‐3 M0 or T1N1‐3 M0 hypopharyngeal squamous cell carcinoma (HPSCC) and the use of postoperative radiotherapy with or without systemic therapy for patients with T1‐2N1M0 HPSCC remain controversial. We aimed to determine whether these additional treatments improve the prognosis in HPSCC.

**Methods:**

We retrospectively analyzed the databases held by the SEER (surveillance, epidemiology, and end results) program and a tertiary referral center in China to evaluate the survival outcomes of surgical intervention for T2‐3N0‐3 M0 and T1N1‐3 M0 HPSCC and of postoperative radiotherapy for T1‐2N1M0 disease.

**Results:**

The SEER contained data for 1235 patients with T2‐3N0‐3 M0 or T1N1‐3 M0 HPSCC, of whom 220 underwent surgery as their first treatment and 737 received non‐surgical treatment. There was no statistically significant difference in overall survival (OS) between these two groups. Data were also available for 30 patients in the SEER who were treated by surgery alone (*n* = 11), surgery plus postoperative radiotherapy (*n* = 7), or surgery plus postoperative radiotherapy with systemic therapy (*n* = 12). Similarly, 23 patients at our hospital were identified to have been treated by surgery alone (*n* = 7), surgery plus postoperative radiotherapy (*n* = 10), or surgery plus postoperative radiotherapy with systemic therapy (*n* = 6). The SEER data indicated that postoperative radiotherapy improved OS (hazard ratio 0.281, 95% confidence interval 0.079–0.998; *p* = 0.036). This finding was supported by the data from our hospital, although the improvement in OS was not statistically significant (hazard ratio 0.360, 95% confidence interval 0.057–2.261; *p* = 0.224). Postoperative radiotherapy with systemic therapy seemed not to improve OS beyond that achieved by postoperative radiotherapy alone.

**Conclusions:**

There was no significant difference in OS in patients with T2‐3N0‐3 M0 or T1N1‐3 M0 HPSCC according to whether or not they underwent surgery as first‐line treatment. Surgery plus postoperative radiotherapy was associated with a more favorable prognosis than surgery alone in patients with T1‐2N1M0 HPSCC.

## 1 Introduction

Hypopharyngeal squamous cell carcinoma (HPSCC) comprises 3%–5% of all cases of head and neck cancer, which is the seventh most common cancer worldwide and has a high incidence in China because of high rates of tobacco smoking and alcohol consumption [1, 2, 3]. Mostly located in the piriform fossa, HPSCC is very aggressive and characterized by submucosal spread, invasion of nerves and blood vessels, a high risk of spread to the lymph nodes, and distant metastasis [4, 5]. The occult nature of primary HPSCC means that the disease often does not present until it is locoregionally advanced [6]. Despite continuous improvements in diagnosis and treatment, HPSCC continues to have the worst prognosis of all the head and neck cancers [7]. Therefore, further comparative clinical research is needed to allow selection of more personalized and effective treatment.

According to the latest National Cancer Comprehensive Network (NCCN) Guidelines for Head and Neck Cancers (version 2.2025; https://www.nccn.org/guidelines/nccn‐guidelines), the initial treatments recommended for patients with T2‐3N0‐3 M0 or T1N1‐3 M0 HPSCC include induction chemotherapy, surgery, and concurrent systemic therapy (ST)/radiotherapy (RT). However, there have been few adequate randomized clinical comparisons of the outcomes of these treatments, which could be helpful to determine which patients would benefit most from which treatment. Furthermore, some clinicians recommend consideration of postoperative RT in patients with T1‐2N1M0 disease based on little clear scientific evidence [8]. The surveillance, epidemiology, and end results (SEER) program, a large‐scale database held by the National Cancer Institute, has accumulated substantial clinical evidence on HPSCC and can provide an adequate number of cases for further analysis [9, 10].

The aims of this study were to determine whether or not surgery is a better treatment for patients with T2‐3N0‐3 M0 or T1N1‐3 M0 HPSCC and whether postoperative radiotherapy could improve the prognosis in those with T1‐2N1M0 HPSCC using data from the SEER program and our hospital database.

## 2 Methods

### 2.1 Data Sources

We extracted data for patients with HPSCC (T2–3 with any N and M0 or T1 with N1–3 and M0) from the SEER database (Nov 2022 Sub, 1975–2020 varying) using SEER*Stat version 8.4.2 software (http://seer.cancer.gov). Patients who underwent partial laryngopharyngectomy with neck lymph node dissection as their first treatment between January 2016 and October 2022 at our tertiary referral center and were diagnosed to have T1‐2N1M0 HPSCC were retrospectively identified and enrolled in the study. None of the patients enrolled from our center had adverse pathologic features (i.e., extranodal extension, positive margins, close margins, pT3 or pT4 primary, pN2 or pN3 nodal disease, perineural invasion, vascular invasion, or lymphatic invasion). All these patients were classified as a low‐to‐intermediate risk group that may benefit from postoperative RT or chemoradiotherapy for disease control according to the NCCN guidelines. The hospital component of the study was approved by our institutional research ethics board with a waiver of the need for informed consent because only retrospective de‐identified data were used. The SEER data are publicly available, so there was no need for ethical approval or informed consent.

### 2.2 Treatment Details

All patients with HPSCC in our hospital cohort underwent partial laryngopharyngectomy with preservation of laryngeal function and ipsilateral or bilateral neck lymph node dissection. None of these patients underwent total laryngectomy or free flap reconstruction. When resection was limited and there was minimal trauma, the remaining mucosa was sutured to repair the hypopharynx. When the surgical defect was extensive, the hypopharyngeal mucosa was covered using artificial dermis. The gastric tube was successfully removed in all cases and performed swallowing exercises 1–2 weeks after surgery, as confirmed by the results of electronic laryngoscopy and esophagography. On the basis of the preoperative imaging examinations and postoperative pathology reports, all patients were finally diagnosed to have T1‐2N1M0 HPSCC by the same team of experienced surgeons (ZH, WL, and WW) according to the Eighth Edition American Joint Committee on Cancer Staging Manual. None of the patients had any adverse pathologic features (i.e., extranodal extension, positive margins, close margins, pT3 or pT4 primary, pN2 or pN3 nodal disease, perineural invasion, vascular invasion, or lymphatic invasion). Some patients received postoperative RT with or without concurrent platinum‐based ST at 4 weeks after surgery.

### 2.3 Follow‐Up

The study outcome was overall survival (OS), which was defined as survival until death from any cause. Cancer‐specific death was not used because information on cause of death is not complete in the SEER database. Our research assistant followed up the patients who underwent surgery at our hospital every 3 months for the first year and every 6 months thereafter until March 2024.

### 2.4 Statistical Analysis

Differences between groups were compared using the chi‐squared test. The two‐tailed *t*‐test assuming equal variances was used for comparison between two groups. If the *p*‐value for the *F* test used to compare variances was < 0.05, Welch's correction was used. The Mann–Whitney test was used to examine non‐normally distributed continuous data. Data for multiple groups were compared by one‐way analysis of variance. If the results of analysis of variance indicated significant differences, post hoc analysis was performed using the Tukey test. OS and median survival time (MST) were compared between groups by the log‐rank test using the survival (version 3.3.1), survminer (version 0.4.9), and ggplot2 (version 3.3.6) packages in R. The statistical analysis was performed using GraphPad Prism 8 (GraphPad Software Inc., San Diego, CA, USA) and R version 4.2.1 (www.r‐project.org). A *p*‐value < 0.05 was considered statistically significant.

## 3 Results

### 3.1 Patient Characteristics

**FIGURE 1:**
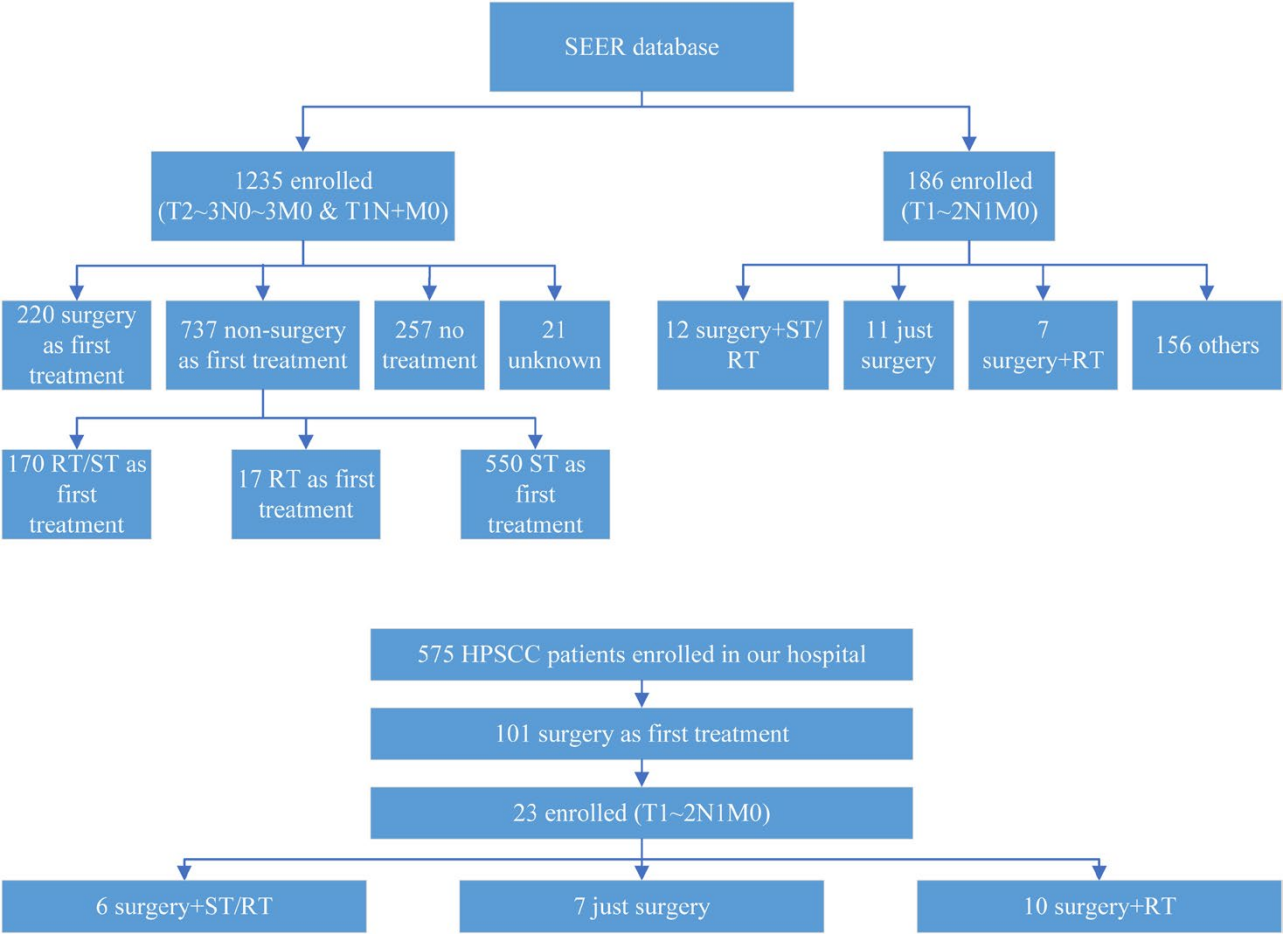
Flowchart showing the procedure used to select data from the SEER database, HPSCC, hypopharyngeal squamous cell carcinoma; RT, radiotherapy; SEER, surveillance, epidemiology, and end results; ST, systemic therapy.

Among the 1235 patients with HPSCC (T2–3 with any N and M0 or T1 with N1–3 and M0) who had data in the SEER database, 220 underwent surgery as the first treatment and 737 received other treatment. Table 1 summarizes the demographics and tumor characteristics in these patients. Eleven patients with T1‐2N1M0 HPSCC in the SEER database underwent surgery alone and seven underwent surgery with postoperative RT (Table 2). Additionally, 12 patients with T1‐2N1M0 HPSCC in the SEER database underwent surgery with postoperative ST/RT (Table S1). Among the 23 patients who underwent surgery as their first treatment for T1‐2N1M0 HPSCC at our hospital during the study period, 30.43% underwent surgery alone, 43.48% underwent surgery with postoperative RT, and 26.09% underwent surgery with postoperative ST/RT (Table 3). The procedure used to select data from the SEER database and our hospital records is shown in Figure 1.

**TABLE 1:** Baseline characteristics of eligible patients identified in the SEER database.

Parameter	All patients	Surgery as first treatment	Non‐surgery as first treatment	Surgery vs. non‐surgery	*p*
Number	1235	220	737		
Sex				χ^2^ = 0.228	0.633
Male	963	177	582		
Female	272	43	155		
Age				*t* = 2.566	0.011
Mean	65.4	65.6	63.6		
Range	23 ~ 90	26 ~ 89	36 ~ 90		
T‐stage				*χ* ^2^ = 1.162	0.559
T1	123	30	81		
T2	694	117	402		
T3	418	73	254		
N‐stage				*χ* ^2^ = 21.134	< 0.001
N0	367	81	160		
N1	261	41	164		
N2	529	85	364		
N3	61	10	43		
Unknown	17	3	6		
Grade				*χ* ^2^ = 11.037	0.012
G1	42	12	20		
G2	420	81	245		
G3	361	85	198		
G4	16	9	6		
Unknown	396	33	268		

Abbreviation: SEER, surveillance, epidemiology, and end results.

**TABLE 2:** Baseline characteristics of SEER patients according to whether or not they received postoperative RT.

Parameter	Surgery	Surgery+RT	*t* or *χ* ^2^	*p*
Number	11	7		
Sex			*χ* ^2^ = 0.004	0.950
Male	8	5		
Female	3	2		
Age			*t* = 0.451	0.662
Mean	66.1	64.0		
Range	56 ~ 79	48 ~ 77		
T‐stage			*χ* ^2^ = 1.169	0.280
T1	6	2		
T2	5	5		
Grade			*χ* ^2^ = 2.109	0.348
G2	5	3		
G3	2	4		
G4	1	0		
Unknown	3	0		

Abbreviations: RT, radiotherapy; SEER, surveillance, epidemiology, and end results.

**TABLE 3:** Baseline characteristics of patients from our hospital.

Parameter	Surgery	Surgery+RT	Surgery+ST/RT	Surgery vs. surgery+RT		Surgery vs. surgery+RT vs. surgery+ST/RT	
				*t* or *χ* ^2^	*p*	*F* or *χ* ^2^	*p*
Number	7	10	6				
Sex				1.518	0.218	1.708	0.426
Male	6	10	5				
Female	1	0	1				
Age				0.815	0.432	0.765	0.478
Mean	61.3	64.6	60.0				
Range	48 ~ 72	53 ~ 76	55 ~ 73				
T‐stage				0.093	0.760	2.477	0.290
T1	1	2	3				
T2	6	8	3				
Grade				0.168	0.682	7.258	0.123
G1	0	0	2				
G2	5	8	2				
G3	2	2	2				

### 3.2 No Difference in OS Between the Surgical and Non‐Surgical Groups

**FIGURE 2:**
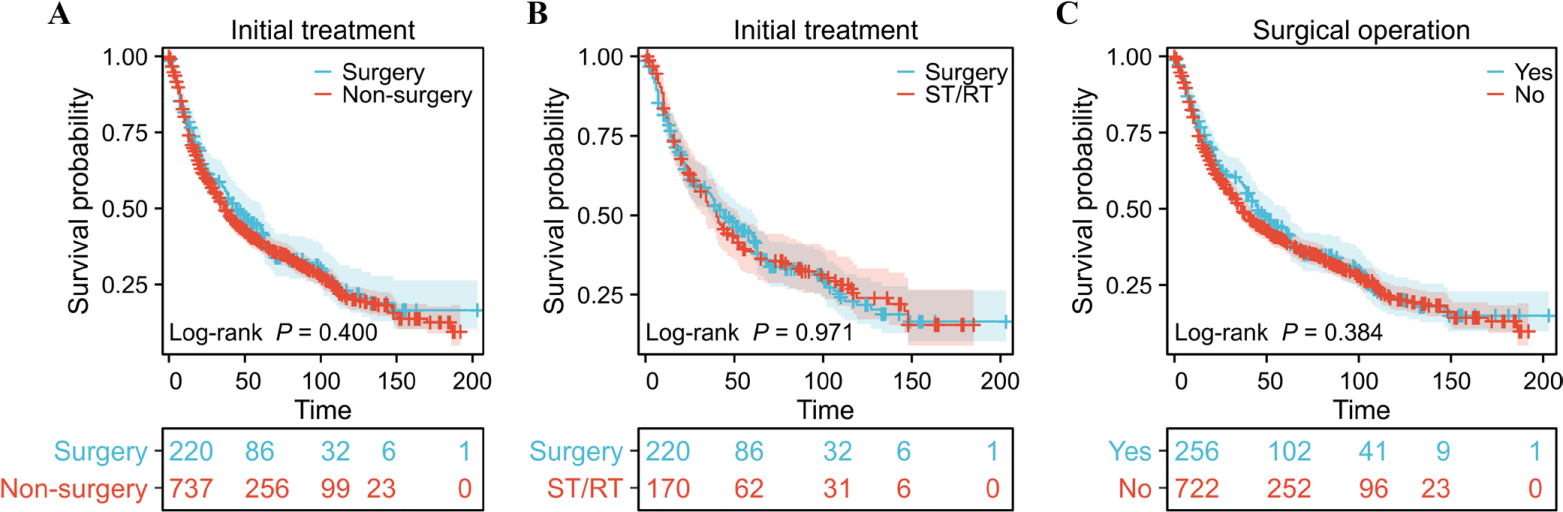
Association between surgery and overall survival in patients with T2‐3N0‐3 M0 or T1N1‐3 M0 hypopharyngeal squamous cell carcinoma. (A) Graph showing no significant difference in overall survival according to whether the initial treatment was surgical or non‐surgical (hazard ratio 1.081, 95% confidence interval 0.903–1.295; *p* = 0.400). (B) Graph showing no significant difference in overall survival according to whether the initial treatment was surgery alone or surgery with concurrent ST/RT (hazard ratio 0.995, 95% confidence interval 0.780–1.270; *p* = 0.971). (C) Graph showing no significant difference in overall survival according to the stage at which surgery was performed (hazard ratio 1.079, 95% confidence interval 0.911–1.277; *p* = 0.384). RT, radiotherapy; ST, systemic therapy.

First, we compared OS between the patients in the SEER database (T2–3 with any N and M0 or T1 with N1–3 and M0) according to whether the initial treatment was surgical (MST 45 months) or non‐surgical (MST 37 months). We found no significant difference in OS between these two groups (hazard ratio [HR] 1.081, 95% confidence interval [CI] 0.903–1.295, *p* = 0.400, log‐rank test; Figure 2A, Table 1) or between patients who underwent surgery (MST 45 months) and those who received concurrent ST/RT (MST 40 months) as the initial treatment (HR 0.995, 95% CI 0.780–1.270; *p* = 0.971, log‐rank test; Figure 2B, Table S2). We also found no significant difference in OS according to whether or not patients underwent surgery at any stage of their treatment (surgery, MST 45 months; non‐surgery, MST 36 months; HR 1.079, 95% CI 0.911–1.277; *p* = 0.384, log‐rank test; Figure 2C, Table S3).

### 3.3 Postoperative RT Improved OS in Patients With T1‐2N1M0 HPSCC

**FIGURE 3:**
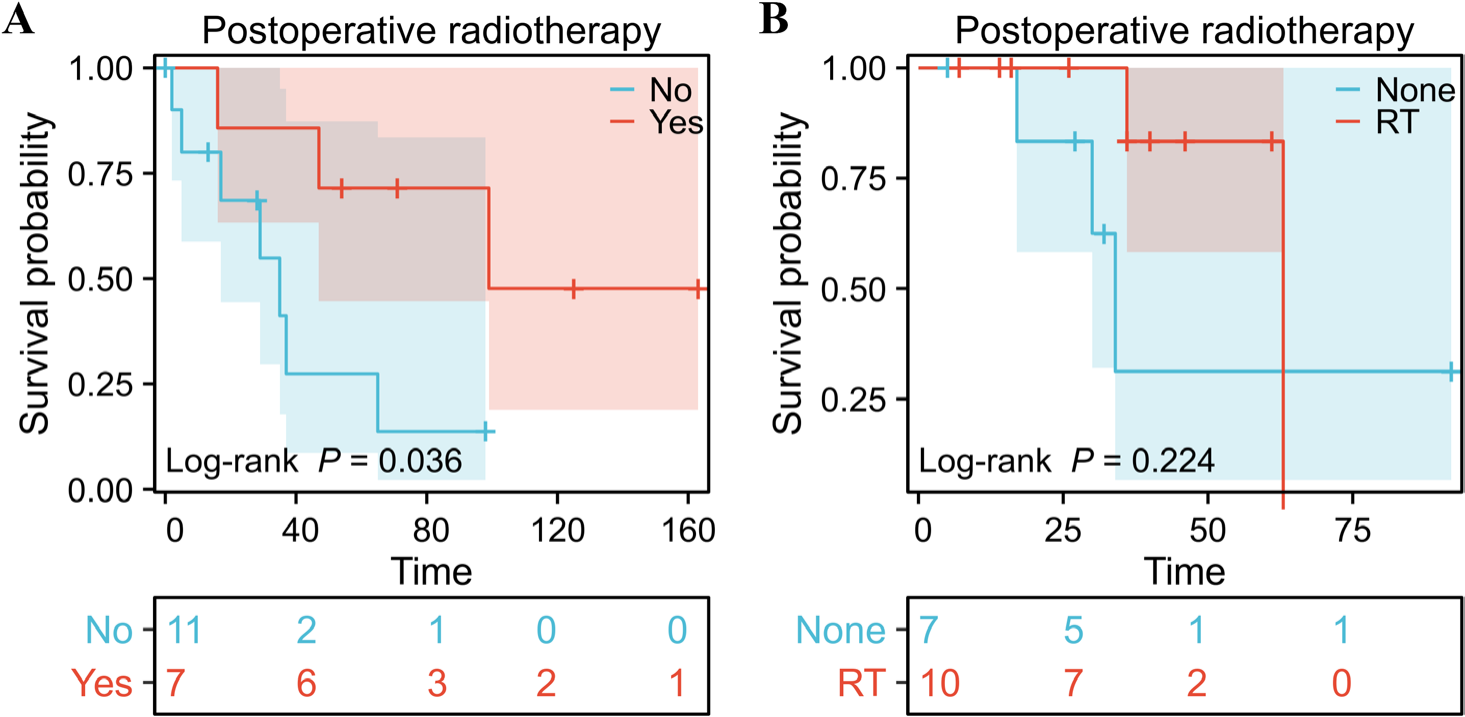
Association between postoperative radiotherapy and overall survival in patients with T1‐2N1M0 hypopharyngeal squamous cell carcinoma. (A) Graph showing that overall survival was better in the SEER group that underwent surgery with postoperative radiotherapy than in the SEER group that underwent surgery alone (hazard ratio 0.281, 95% confidence interval 0.079–0.998; *p* = 0.036). (B) Data from our hospital showed that overall survival was better in the group that received postoperative radiotherapy, albeit a finding that was not statistically significant (hazard ratio 0.360, 95% confidence interval 0.057–2.261; *p* = 0.224). SEER, surveillance, epidemiology, and end results; RT, radiotherapy.

Next, we used the data from the SEER database and the patients treated at our hospital to compare the difference in OS between those with T1‐2N1M0 HPSCC who underwent surgery alone and those who underwent surgery with postoperative RT. Analysis of the SEER data revealed that OS was significantly longer in the group that underwent surgery with postoperative RT (MST 99 months) than in the group that underwent surgery alone (MST 35 months; HR 0.281, 95% CI 0.079–0.998; *p* = 0.036, log‐rank test; Figure 3A). At our hospital, OS was also longer in patients who underwent surgery with postoperative RT (MST 63 months) than in those who underwent surgery alone (MST 34 months); the difference did not reach statistical significance (HR 0.360, 95% CI 0.057–2.261; *p* = 0.224, log‐rank test; Figure 3B), possibly because of the small sample size and relatively short follow‐up duration.

### 3.4 No Difference in OS Between Postoperative RT and Postoperative ST/RT in Patients With T1‐2N1M0 HPSCC

**FIGURE 4:**
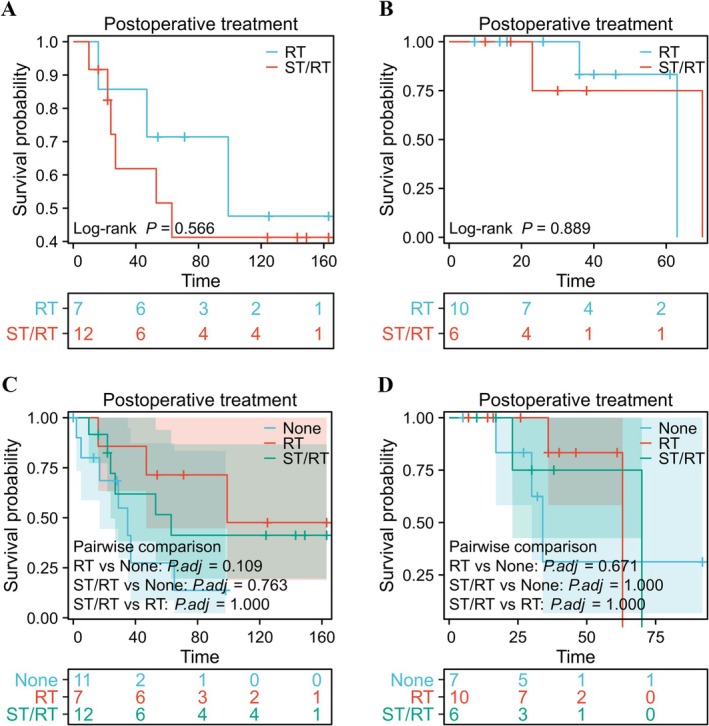
Association between postoperative ST/RT and overall survival in patients with T1‐2N1M0 hypopharyngeal squamous cell carcinoma. (A) Graph showing no significant difference in overall survival in SEER patients according to whether RT or ST/RT was administered postoperatively (hazard ratio 1.495, 95% confidence interval 0.399–5.600; *p* = 0.566). (B) Graph showing no significant difference in overall survival according to whether RT or ST/RT was administered postoperatively in patients from our hospital (hazard ratio 0.892, 95% confidence interval 0.125–6.356; *p* = 0.890). (C) Graph showing no significant difference in overall survival among SEER patients according to whether they received surgery alone, postoperative RT, or postoperative RT/ST (*χ* ^2^ = 3.830, *p* = 0.147). (D) Graph showing no significant difference among patients from our hospital according to whether they received surgery alone, postoperative RT, or postoperative RT/ST (*χ* ^2^ = 0.759, *p* = 0.684). RT, radiotherapy; SEER, surveillance, epidemiology, and end results; ST, systemic therapy.

Finally, to determine whether or not postoperative ST/RT could further improve OS in patients with T1‐2N1M0 HPSCC, we compared the results for postoperative RT alone with those for postoperative ST/RT using the data from the SEER database and our hospital. Analysis of the SEER results revealed that patients who received postoperative ST/RT (MST 63 months) had no survival advantage over those who received postoperative RT alone (MST 99 months) (HR 1.495, 95% CI 0.399–5.600; *p* = 0.566, log‐rank test; Figure 4A). This result was consistent with that for patients at our hospital (ST/RT, MST 70 months; RT alone, MST 63 months; HR 0.892, 95% CI 0.125–6.356; *p* = 0.890, log‐rank test; Figure 4B). Furthermore, there were no statistically significant differences in OS among the patients who received surgery alone, postoperative RT, or postoperative RT/ST in either the SEER data (Figure 4C, *χ* ^2^ = 3.830, *p* = 0.147) or our hospital data (Figure 4D, *χ* ^2^ = 0.759, *p* = 0.684).

## 4 Discussion

At present, the therapeutic management of HPSCC remains controversial and varies widely from center to center [4]. Therapeutic decision‐making for HPSCC is complex and requires multidisciplinary collaboration, given that it often affects breathing, swallowing, and quality of voice [4, 11]. Although organ preservation therapies, including RT and concurrent ST/RT, have often been used in the treatment of primary HPSCC, the recurrence rate has been up to approximately 50%–60% [12, 13]. Furthermore, with the high rate of recurrence or metastasis, the 5‐year OS rate in patients with HPSCC is only 25%–40% [14, 15]. Therefore, surgery has always held an important place in HPSCC, regardless of whether it is used to treat the primary tumor, a recurrence, or metastasis [16, 17].

Our analysis of the SEER data found no significant difference in OS between patients with HPSCC (T2–3 with any N and M0 or T1 with N1–3 and M0) according to whether they underwent surgery, were treated non‐surgically, or received concurrent ST/RT as the initial treatment. We also found that there was no significant difference in OS between patients who underwent surgery at any stage of their treatment and those who did not, which is consistent with another report [18].

Currently, standard management for HPSCC comprises ST with multiagent chemotherapy and local control with surgery and RT [19, 20]. The main advantages of surgery are its better local control rate, longer progression‐free survival [16, 21], and relatively low cost; however, these benefits come at the cost of unwanted effects, including hoarseness of voice, difficulty swallowing, and pharyngeal fistula, which may have a severe impact on the patient's quality of life [7, 22, 23]. In contrast, ST can preserve not only the functional status of the organ but also that of the hypopharynx, but is associated with a high long‐term recurrence rate and systemic toxicity that cause difficulties for survivors [24, 25, 26]. In our opinion, the lack of more detailed data in the SEER database, including for surgical techniques, postoperative voice quality, swallowing function, and other functional outcomes, precludes any definitive conclusions regarding the comparative therapeutic advantages of the various treatment modalities. Further prospective clinical investigations that incorporate multidimensional assessments are warranted to establish optimal management strategies for patient‐centered decision‐making.

With the development of equipment and technology, RT has emerged as an important organ preservation method for HPSCC [27]. The main RT modalities used in HPSCC are radical RT, adjuvant RT, and concurrent ST/RT. Radical RT is always recommended for primary early‐stage HPSCC [28, 29]. Katano et al. [30] investigated 72 patients with clinical stage I or II HPSCC who underwent radical RT and reported a 5‐year OS rate of 80.7% and a 5‐year disease‐free survival rate of 66.4%. Their findings provide evidence for radical RT being an effective approach for the management of early‐stage HPSCC. However, for patients with locally advanced HPSCC, as in our study, radical RT seems not to be a suitable initial treatment and is not recommended in the NCCN guidelines.

Our analysis of the SEER data showed that postoperative RT could improve the MST from 35 months to 99 months in patients with T1‐2N1M0 HPSCC. This finding is consistent with our hospital data, which showed an increase in MST in these patients from 34 to 63 months, albeit not a finding that reached statistical significance. Both surgical treatment and concurrent chemoradiotherapy are essential options for patients with early‐stage HPSCC. A multicenter retrospective study found no significant difference in OS between patients with early‐stage laryngeal or hypopharyngeal cancer according to whether they were treated surgically or non‐surgically [31]. However, one advantage of surgery is its ability to directly remove the tumor, which reduces the risk of local recurrence [4]. Furthermore, surgery typically requires a shorter duration to complete, and postoperative pathological examination of specimens provides clearer information for staging and grading. In contrast, the treatment cycle for concurrent chemoradiotherapy is longer, requiring patients to undergo treatment over an extended period, which may lead to decreased compliance with treatment. Moreover, advances in surgical techniques, especially in robotic surgery, are resulting in distinct advantages of surgery over RT in terms of OS for patients with early‐stage hypopharyngeal cancer, whether provided alone or in combination with chemotherapy [32].

Laryngeal‐preserving surgery followed by adjuvant RT is an essential therapy for patients with HPSCC and any adverse pathologic features, such as extranodal extension, positive margins, close margins, pT3 or pT4 primary, pN2 or pN3 nodal disease, perineural invasion, vascular invasion, and lymphatic invasion [33]. Some clinicians recommend “Consider RT” in patients with T1‐2N1M0 HPSCC who have undergone radical resection, but in the absence of adequate evidence based on high‐quality clinical research.

Whether or not a patient with head and neck cancer will benefit from postoperative RT is always a concern [34, 35, 36]. Frank et al. investigated 110 patients with clinical stage I–IV HPSCC and found that the 5‐year OS rate was significantly higher in those who underwent surgery with postoperative RT than in those who underwent surgery alone (18% vs. 48%) [37]. In another study, Tao et al. found that low‐dose postoperative RT (50 Gy, 78 cases) was non‐inferior to high‐dose postoperative RT (60 Gy, 78 cases) in terms of OS, progression‐free survival, locoregional control, and metastasis‐free survival in patients with stage III or IV HPSCC [8]. However, in a case‐matched cohort analysis of patients with locally advanced head and neck squamous cell carcinoma who were followed up for a median of 40 months, Ren et al. found no significant difference in OS or disease‐specific survival between the group that underwent surgery alone and the group that underwent surgery plus postoperative RT [38]. To our knowledge, the present study is the first to find that postoperative RT without ST can significantly improve OS in patients with T1‐2N1M0 HPSCC. Prospective high‐quality clinical studies are now needed to confirm this finding.

Our finding that a proportion of patients with T1‐2N1M0 HPSCC received postoperative ST/RT has aroused our interest in the prognostic difference between postoperative RT alone and postoperative ST/RT has aroused our interest in the prognostic difference between postoperative RT alone and postoperative ST/RT. Both treatments would inevitably worsen the functional outcomes, and the survival benefit of postoperative ST/RT is not well established [4]. Our results for patients in the SEER database and those treated at our hospital indicate that postoperative ST/RT seems not to improve OS beyond that achieved by postoperative RT alone. However, further studies in larger groups of patients are needed to confirm this impression.

Despite our efforts to ensure data accuracy and statistical robustness, this study has some limitations. First, its retrospective nature introduces the possibility of selection bias and confounding factors. Second, confinement to a public database and a single center limits the generalizability of our results. Third, small numbers of patients with T1‐2N1M0 HPSCC underwent primary surgery with and without postoperative RT or ST/RT. Finally, induction chemotherapy, which is another important treatment, was not investigated.

## 5 Conclusions

Based on the findings of this study, we conclude that there is no significant difference in OS between patients with T2‐3N0‐3 M0 or T1N1‐3 M0 HPSCC who are treated surgically and those who are treated non‐surgically, that postoperative RT can improve OS in patients with T1‐2N1M0 HPSCC, and that postoperative ST/RT seems not to improve OS beyond that achieved by postoperative RT alone. Our findings offer actionable insights regarding the management of these patients. Nevertheless, prospective multicenter clinical studies are required to validate and refine these findings and pave the way for optimized therapeutic strategies.

## Author Contributions

Conceptualization: Zhangwei Hu, Weiping Wen, Wenbin Lei. Methodology: Zhangwei Hu, Renqiang Ma, Yihui Wen, Wei Sun, Siyu Chen. Software: Zhangwei Hu, Renqiang Ma, Jie Deng, Wei Sun, Lin Chen. Data curation: Zhangwei Hu, Renqiang Ma, Lin Chen. Investigation: Zhangwei Hu, Renqiang Ma, Yihui Wen, Jie Deng. Validation: Renqiang Ma, Jie Deng, Lin Chen, Siyu Chen. Formal analysis: Zhangwei Hu, Yihui Wen, Jie Deng, Wei Sun, Siyu Chen. Supervision: Yihui Wen, Wei Sun, Lin Chen, Weiping Wen, Wenbin Lei. Funding acquisition: Zhangwei Hu, Wenbin Lei. Visualization: Renqiang Ma, Wei Sun, Siyu Chen. Project administration: Yihui Wen, Lin Chen, Siyu Chen. Resources: Yihui Wen, Wei Sun, Weiping Wen, Wenbin Lei. Writing – original draft: Zhangwei Hu. Writing – review and editing: Zhangwei Hu, Jie Deng, Weiping Wen, Wenbin Lei.

## Funding

This study was supported by the National Natural Science Foundation of China (82403372), Guangzhou Basic and Applied Basic Special Research Project Young PhD “Qi Hang” Foundation (2025A04J4018), GuangDong Basic and Applied Basic Research Foundation (2022A1515110147), China Postdoctoral Science Foundation (2021 M703712), National Key R&D Program of China (2020YFC1316903), and 5010 Clinical Research Program of Sun Yat‐sen University (2017004).

## Ethics Statement

The hospital component of the study was approved by our institutional research ethics board with a waiver of the need for informed consent because only retrospective de‐identified data were used. The SEER data are publicly available, so this component of the study did not require ethical approval or informed consent.

## Conflicts of Interest

The authors declare no conflicts of interest.

## Supporting information


**Table S1:** Baseline characteristics of SEER patients according to type of postoperative treatment.

**Table S2:** Baseline characteristics of SEER patients according to whether they received surgery or ST/RT as initial treatment.

**Table S3:** Baseline characteristics of SEER patients according to whether or not they underwent surgery.

## Data Availability

The datasets used and/or analyzed during the current study are available from the corresponding author on reasonable request.
