# Supplementary material for: Survival Benefit of Adjuvant Radiotherapy After Surgery in Patients With T1‐2N1M0 Hypopharyngeal Squamous Cell Carcinoma: A Dual‐Cohort Analysis of SEER and Institutional Data
Source: Cancer Med. 2026 Jan 30;15(2):e71555. doi: 10.1002/cam4.71555 (PMC12856699; doi:10.1002/cam4.71555)
Supplement: Supplementary file 1 — Table S1: Baseline characteristics of SEER patients according to type of postoperative treatment. [file CAM4-15-e71555-s003.docx]

**Supplementary table 1.** **Baseline characteristics of SEER patients according to type of postoperative treatment**

| Parameter | Surgery | Surgery+RT | Surgery+ST/RT | F or χ2 | p-value |
| --- | --- | --- | --- | --- | --- |
| Number | 11 | 7 | 12 |  |  |
| Sex |  |  |  | χ2 = 0.109 | 0.947 |
| Male | 8 | 5 | 8 |  |  |
| Female | 3 | 2 | 4 |  |  |
| Age |  |  |  | F = 0.182 | 0.835 |
| Mean | 66.1 | 64.0 | 63.9 |  |  |
| Range | 56~79 | 48~77 | 46~78 |  |  |
| T-stage |  |  |  | χ2 = 1.198 | 0.549 |
| T1 | 6 | 2 | 5 |  |  |
| T2 | 5 | 5 | 7 |  |  |
| Grade |  |  |  | χ2 = 5.788 | 0.447 |
| G1 | 0 | 0 | 1 |  |  |
| G2 | 5 | 3 | 2 |  |  |
| G3 | 2 | 4 | 3 |  |  |
| G4 | 1 | 0 | 0 |  |  |
| Unknown | 3 | 0 | 6 |  |  |
