# Supplementary material for: Survival Benefit of Adjuvant Radiotherapy After Surgery in Patients With T1‐2N1M0 Hypopharyngeal Squamous Cell Carcinoma: A Dual‐Cohort Analysis of SEER and Institutional Data
Source: Cancer Med. 2026 Jan 30;15(2):e71555. doi: 10.1002/cam4.71555 (PMC12856699; doi:10.1002/cam4.71555)
Supplement: Supplementary file 2 — Table S2: Baseline characteristics of SEER patients according to whether they received surgery or ST/RT as initial treatment. [file CAM4-15-e71555-s001.docx]

**Supplementary table 2. Baseline characteristics of SEER patients according to whether they received surgery or** **ST/RT as initial treatment**

| Parameter | Surgery as first treatment | ST/RT as first treatment | t or χ2 | p-value |
| --- | --- | --- | --- | --- |
| Number | 220 | 170 |  |  |
| Sex |  |  | χ2 = 0.290 | 0.590 |
| Male | 177 | 133 |  |  |
| Female | 43 | 37 |  |  |
| Age |  |  | t = 1.577 | 0.116 |
| Mean | 65.6 | 64.0 |  |  |
| Range | 26~89 | 37~87 |  |  |
| T-stage |  |  | χ2 = 9.234 | 0.010 |
| T1 | 30 | 34 |  |  |
| T2 | 117 | 102 |  |  |
| T3 | 73 | 34 |  |  |
| N-stage |  |  | χ2 = 60.667 | <0.001 |
| N0 | 81 | 9 |  |  |
| N1 | 41 | 29 |  |  |
| N2 | 85 | 114 |  |  |
| N3 | 10 | 17 |  |  |
| Unknown | 3 | 1 |  |  |
| Grade |  |  | χ2 = 2.505 | 0.474 |
| G1 | 12 | 3 |  |  |
| G2 | 81 | 40 |  |  |
| G3 | 85 | 46 |  |  |
| G4 | 9 | 2 |  |  |
| Unknown | 33 | 79 |  |  |
