# Supplementary material for: Survival Benefit of Adjuvant Radiotherapy After Surgery in Patients With T1‐2N1M0 Hypopharyngeal Squamous Cell Carcinoma: A Dual‐Cohort Analysis of SEER and Institutional Data
Source: Cancer Med. 2026 Jan 30;15(2):e71555. doi: 10.1002/cam4.71555 (PMC12856699; doi:10.1002/cam4.71555)
Supplement: Supplementary file 3 — Table S3: Baseline characteristics of SEER patients according to whether or not they underwent surgery. [file CAM4-15-e71555-s002.docx]

**Supplementary table 3. Baseline characteristics of SEER patients according to whether or not they underwent surgery**

| Parameter | With surgery | Without surgery | t or χ2 | p-value |
| --- | --- | --- | --- | --- |
| Number | 256 | 722 |  |  |
| Sex |  |  | χ2 = 0.226 | 0.635 |
| Male | 205 | 568 |  |  |
| Female | 51 | 154 |  |  |
| Age |  |  | t = 2.050 | 0.041 |
| Mean | 65.2 | 63.6 |  |  |
| Range | 26~89 | 36~90 |  |  |
| T-stage |  |  | χ2 = 0.426 | 0.808 |
| T1 | 32 | 81 |  |  |
| T2 | 141 | 395 |  |  |
| T3 | 83 | 246 |  |  |
| N-stage |  |  | χ2 = 19.477 | <0.001 |
| N0 | 91 | 157 |  |  |
| N1 | 49 | 161 |  |  |
| N2 | 101 | 357 |  |  |
| N3 | 12 | 41 |  |  |
| Unknown | 3 | 6 |  |  |
| Grade |  |  | χ2 = 6.985 | 0.072 |
| G1 | 12 | 20 |  |  |
| G2 | 100 | 237 |  |  |
| G3 | 98 | 192 |  |  |
| G4 | 9 | 6 |  |  |
| Unknown | 37 | 267 |  |  |
